# Co-design and development of a Personalised Exercise-based Rehabilitation and self-management programme FOR people with Multiple long-term conditions: The PERFORM intervention

**DOI:** 10.1177/26335565251367326

**Published:** 2025-09-18

**Authors:** Paulina Daw, Colin J. Greaves, Nikki Gardiner, Patrick Doherty, Thomas M. Withers, Amy C. Barradell, Paul O’Halloran, Zahira Ahmed, Shaun Barber, Gwen Barwell, Sophie E. Brown, Sarah Dean, Carlos Echevarria, Rachael A. Evans, Tracy Ibbotson, Bhautesh D. Jani, Kate Jolly, James R. Manifield, Frances S. Mair, Emma McIntosh, Daniel Miller, Paula Ormandy, Susan M. Smith, Sharon A. Simpson, Ghazala Waheed, Rod S. Taylor, Sally J. Singh, on behalf of the PERFORM research team

**Affiliations:** 1 School of Sport, Exercise and Rehabilitation Sciences, 1724 University of Birmingham, Birmingham, UK; 2 Department of Cardiopulmonary Rehabilitation, 4490 University Hospitals of Leicester NHS Trust, Leicester, UK; 3 Department of Health Science, 66134 University of York, York, UK; 4 School of Psychology and Vision Sciences, University of Leicester, Leicester, UK; 5 Centre for Sport and Social Impact, 622931 La Trobe University, Melbourne, Victoria, Australia; 6 School of Psychology and Public Health, 622931 La Trobe University, Melbourne, Victoria, Australia; 7 NIHR Biomedical Research Centre, Department of Respiratory Sciences, 574216 University of Leicester, Leicester, UK; 8 Clinical Trials Unit, 487969 University of Leicester, Leicester, UK; 9 MRC/CSO Social and Public Health Sciences Unit, School of Health and Wellbeing, 47970 University of Glasgow, Glasgow, UK; 10 171002 University of Exeter Medical School, Exeter, UK; 11 5983 Newcastle Upon Tyne Hospitals NHS Foundation Trust, Newcastle upon Tyne, UK; 12 Translational and Clinical Research Institute, Newcastle University, Newcastle upon Tyne, UK; 13 General Practice and Primary Care, School of Health and Wellbeing, 347493 University of Glasgow, Glasgow, UK; 14 Department of Applied Health Sciences, 1724 University of Birmingham, Birmingham, UK; 15 Respiratory Sciences, 574216 University of Leicester, Leicester, UK; 16 Health Economics and Health Technology Assessment, School of Health and Wellbeing, 574487 University of Glasgow, Glasgow, UK; 17 The Reach for Health Centre, Daventry, UK; 18 School of Health and Society, 105168 University of Salford, Manchester, UK; 19 Department of Public Health and Primary Care, 8809 Trinity College Dublin, Dublin, Ireland; 20 Robertson Centre for Biostatistics, School of Health and Wellbeing, 47970 University of Glasgow, Glasgow, UK

**Keywords:** intervention development, multiple long-term conditions, exercise, self-management, health-related quality of life, rehabilitation, multimorbidity, behaviour change

## Abstract

**Background:**

Exercise and self-management support may be clinically effective and cost-effective treatments for a range of individual long-term conditions (LTCs), as they activate multiple beneficial physiological and psychological mechanisms. We aimed to develop a complex intervention to deliver exercise and self-management support for people with multiple LTCs (MLTCs).

**Methods:**

Following the Person Based Approach to intervention development, we conducted ten co-development workshops with people with MLTCs, family and friends; healthcare providers; service commissioners and policymakers. The workshops iteratively identified the unmet needs of people with MLTCs and informed a programme theory outlining theoretical mechanisms of change and intervention strategies to change the targeted behaviours. They also identified ideas for efficient delivery and service providers’ training needs. Mixed methods feedback from the Personalised Exercise-Rehabilitation FOR people with Multiple long-term conditions (PERFORM) feasibility study (reported elsewhere) informed intervention refinement.

**Results:**

A diverse group of stakeholders (26 people with MLTCs/supporters, 13 service providers, 16 experts in chronic illness and 14 service commissioners) helped to develop the PERFORM intervention. This included 16 supervised exercise sessions and 16 ‘Health and Wellbeing’ self-management support sessions, delivered in hospital or community settings over eight weeks, plus check-in sessions at four and six months. The self-management sessions covered maintenance of exercise/physical activity, healthy eating and managing common symptoms (pain, fatigue, breathlessness, stress).

**Conclusion:**

The PERFORM intervention is a comprehensive, evidence-informed, theoretically driven self-management and exercise-based rehabilitation intervention, co-developed with people with MLTCs, service providers and service commissioners. PERFORM is now ready for evaluation regarding clinical effectiveness and cost-effectiveness.

## Background

Improving healthcare for people with multiple long-term conditions (MLTCs), or ‘multimorbidity’, is a key challenge for healthcare systems in the UK and globally. The prevalence of MLTCs is increasing^1^ due to aging populations, suboptimal health behaviours, and socioeconomic deprivation.^2,3^ The burden of MLTCs to patients, carers, healthcare systems and society is high due to reduced health-related quality of life, functional decline, increased mortality and increased healthcare utilisation.^4–7^

People with MLTCs may present with a wide range of conditions and the aetiology, severity and treatments can be vastly different. Consequently, more nuanced approaches are needed to identify clusters of LTCs with common aetiologies that could benefit from similar treatment approaches.^8^ Interventions aimed at such clusters may potentially increase the efficiency of health services and reduce treatment burden for people living with MLTCs.^9–11^

A 2021 Cochrane systematic review and meta-analysis found few effective interventions for MLTCs self-management.^12^ In contrast, there is a substantial effectiveness evidence for disease-specific self-management interventions to improve functional capacity, health-related quality of life, and reduce hospital admissions for conditions including heart disease, arthritis, respiratory disease, stroke and several cancers. In particular, exercise-based rehabilitation programmes have been shown to be both effective (leading to improvement in functional capacity, health-related quality of life and reduced hospital admissions) and cost-effective for cardiac or pulmonary conditions.^13,14^ In the UK, National Health Service (NHS) exercise-based rehabilitation services are available for several index conditions (e.g., pulmonary disease, cardiovascular disease and long COVID) in hospitals and community centres, as well as some home-based programmes.^15^ Recent evidence from Denmark shows that a twelve-week exercise and self-care support programme for people with two or more selected LTCs significantly improved quality of life (measured by EQ-5D) at one year of follow-up.^11^

In the UK, pulmonary and cardiac rehabilitation programmes, which include aerobic activity and resistance exercise, may provide a good starting point for an intervention that addresses MLTCs. These programmes improve many of the symptoms commonly experienced by people with MLTCs, such as functional limitations, breathlessness, pain or low mood.^16–19^ As well as exercise, most existing rehabilitation programmes have been designed to deliver self-management support,^20^ which can address common physical and emotional symptoms such as pain, fatigue, low mood and emotional distress.^21^

Rehabilitation programmes to date have focused on treating a specified ‘index’ LTC (e.g., heart failure or chronic obstructive pulmonary disease). This siloed approach limits access for people with other LTCs, increases the treatment burden for people who are eligible for multiple rehabilitation programmes, may lead to duplication of effort and conflicting advice, and may neglect the more complex needs of people living with MLTCs.

The Personalised Exercise-Rehabilitation FOR people with Multiple long-term conditions (PERFORM) is a research collaboration and programme funded by the National Institute of Health Research that seeks to develop and evaluate a comprehensive exercise-based rehabilitation and self-management support intervention specifically designed for people with MLTCs, for use in the UK’s NHS.^22^ In this paper, we describe the development of the PERFORM intervention.

## Methods

### Study design

The methods are reported in accordance with the GUIDance for the rEporting of intervention Development (GUIDED) checklist (Appendix 1)^23^ and the Template for Intervention Description and Replication (TIDieR) checklist (Appendix 2).^24^ The intervention development process was conducted in accordance with the updated Medical Research Council guidance for developing complex interventions.^25^

We used the Person Based Approach to intervention development, which seeks to optimise effectiveness by matching intervention strategies to barriers and enabling factors identified by multiple relevant partners (e.g., people with MLTCs, healthcare providers).^26^ The Person Based Approach also aims to maximise engagement of the partners to ‘gain vital insights into how different people experience and implement interventions’ and ‘identify the key characteristics that will make an intervention more meaningful, attractive, and useful to those who engage with it’.^26^ In applying the Person Based Approach, we drew on expert opinion, existing evidence, best practice in cardiac and pulmonary rehabilitation and close collaboration with people with MLTCs, caregivers, clinicians and service commissioners.

### Identifying the target population

We conducted three meta-analytic data science studies to identify co-occurring clusters of LTCs with a) the highest negative impact on health outcomes (mortality, hospitalisations, general practice use, and quality of life), b) the greatest healthcare costs in terms of healthcare resource use and c) a confirmed evidence base for benefit from exercise in improving health-related quality of life.^27–29^ The results of these studies informed our initial target population for the PERFORM intervention (see Table 1). Broadly, we targeted adults (aged 18 or over) with two or more LTCs from a list of LTCs which negatively impact health outcomes,^30^ with at least one of their conditions having evidence of benefit from increasing exercise.^31^

**Table 1.** Eligibility criteria for the PERFORM intervention (as used in the PERFORM feasibility study).

Inclusion criteria	Exclusion criteria
Adults ≥18 years old Two or more LTCs, with a) at least one from the following list of LTCs with evidence of benefits from exercise: Arthritis (rheumatoid or osteoarthritis in any joint), asthma, atrial fibrillation, bronchiectasis, cancer, chronic kidney disease, chronic obstructive pulmonary disease, connective tissue disease (pain), coronary heart disease, dementia, depression, diabetes mellitus, heart failure, hypertension, long-COVID, multiple sclerosis, osteoporosis, painful conditions, Parkinson’s disease, peripheral vascular disease, polycystic ovarian syndrome, psychoactive substance misuse, stroke/transient ischaemic attack, alcohol problems, inflammatory bowel disease or prostate disorders b) At least one other LTC from a list of 45 LTCs listed in the Cambridge multimorbidity score^30^ Ambulatory (with walking aids if needed) Able and willing to provide consent	Unable to give consent for treatment Unable to communicate in English (and carer or support worker not available) Known contraindications to exercise as defined by the American College of sports medicine in the ‘11th edition of ACSM’s guidelines for exercise testing and prescription’, to include: - Unstable cardiac disease - Current fever - Significant aortic aneurysm (more than 5.5 cm) Unable to attend in-person exercise sessions Participation in an exercise rehabilitation programme in the last 6 months Unstable psychiatric disorder that limits or disrupts group-based interventions On an end-of-life pathway with a prognosis of less than 12 months survival Active malignancy (on chemotherapy/radiotherapy/planned urgent surgery) For people on a surgical waiting list (a pragmatic decision to be made on a case-by-case basis considering the type of surgery, urgency and likely wait times) Pregnancy Living in a nursing home

### Stakeholder engagement workshops

We engaged with several diverse groups of stakeholders via a series of co-design workshops, aiming to iteratively identify: (a) the unmet needs of people with MLTCs; (b) a behavioural specification (i.e., who needs to do what for the intervention to succeed in engaging people with MLTCs in exercise and self-management behaviours to improve their quality of life?); (c) what processes (barriers and enablers) will influence these behaviours?; (d) strategies for activating the intended intervention processes (intervention techniques); (e) a programme theory and logic model showing how delivery of the intervention will lead to changes in the targeted behaviours and changes in quality of life (the primary target) and other intended outcomes; (f) any necessary changes to existing healthcare delivery systems/service pathways; (g) the training needs of the intended service providers and (h) barriers and enablers to future implementation of the planned service.

With help from the PERFORM Patient and Participant Involvement and Engagement (PPIE) Group, we recruited three stakeholder advisory groups (1): Lived Experience Advisory Group; people living with a diverse range of MLTCs and their supporters (family and friends with substantive input to their everyday care) –; (2) Service Provider Advisory Group; healthcare professionals involved in the delivery of existing care (e.g., cardiac and pulmonary) rehabilitation services, GPs and mental health professionals), and (3) commissioners and policy makers (e.g., representatives from NHS England, key charities from the Richmond Group of Charities, Chartered Society of Physiotherapy, NHS National Clinical Directors and Speciality Advisers). When populating our stakeholder groups, we strove to maximise diversity in terms of diagnosed LTCs, age, gender, ethnicity and cultural background, and geographic area for people with LTCs; and job role/area of expertise for healthcare professionals by employing purposive sampling.^32^ To achieve this, we selected our stakeholders from a pool of volunteers in a way that is mindful of the above characteristics. For example, people with LTCs had to have two or more LTCs that substantially affect their quality of life (we did not specify which conditions, as, at the time, we were still awaiting evidence from our review on which conditions benefit from exercise). If a certain characteristic was underrepresented in our initial sample, for example, we missed a patient from a Black ethnic minority, we contacted our PPIE group to help us with recruitment.

We conducted a series of online (group-based) ‘key informant’ workshops, consisting of: five focus groups with ‘people with MLTCs and supporters/Lived Experience Advisory Group’ (26 members); four focus groups with ‘healthcare professionals’ (13 members); one ‘commissioners and policy makers’ focus group (14 members). Members who were unable to attend these meetings were offered individual phone conversations. Each group-based workshop lasted two hours, was audio recorded and focused on pre-specified aims (Appendix 3).

In parallel to the above workshops, we formed an Expert Advisory Panel consisting of 16 experts in rehabilitation medicine (cardiac and pulmonary), multimorbidity, intervention development and behaviour change, general practice, exercise science/physiology, health economics and statistics, and trial design and evaluation. We created an intervention summary or ‘scaffolding’ document that either specified or asked questions about the target population, intervention aims and targets for change (symptoms, self-management behaviours), intervention content and mode of delivery, implementation contexts (e.g., possible sources of referral, delivery location), likely service providers and staff-training requirements. The document was subjected to several rounds of review by members of the PERFORM expert advisory group, as well as being informed by the workshop activities described above.

A researcher listened to audio recordings after each group workshop and extracted key messages from the narrative; these were circulated to participants for further comments and corrections. We analysed the data (by re-listening to the audio files and note-taking, rather than formal thematic analysis) to extract key behaviour change targets for a) people with MLTCs and carers and b) healthcare professionals. The decision not to conduct a formal thematic analysis was based on the understanding that such a task would be time-consuming and resource-intensive, given the large number of research participants and activities, and would likely not yield significant contributions to the structure or content of the intervention. Furthermore, including a formal data analysis could have distracted us from the main aim of the study: To create an intervention in a timely manner, with the goal that it can be tested in a feasibility study and a trial.

Each uncovered behaviour change target was expanded into relevant sub-behaviours, along with any barriers or enabling factors identified through the workshops or by our Expert Advisory Group input. To help select intervention strategies, we used the Theory and Techniques Tool (an online tool for linking behaviour change techniques and mechanisms of action based on literature synthesis and expert consensus).^33^

Intervention content was further guided by relevant clinical guidance (the British Association for Cardiovascular Prevention and Rehabilitation Standards and Core Components for cardiovascular disease prevention and rehabilitation^34^ and the British Thoracic Society guideline on pulmonary rehabilitation in adults^35^) and vetted information/evidence sources. For example, we reviewed patient information materials from NHS websites to inform specific elements of the programme’s content (e.g., evidence-based methods for managing pain or low mood) and drew on the American College of Sports Medicine guidance on prescribing exercise for people with LTCs to address safety considerations and generate condition-specific advice on exercise where needed.^36^

The target population for the PERFORM intervention (see Table 1) was further refined following a single-arm mixed-methods feasibility study in which 60 patients received the PERFORM intervention (full details of which are reported elsewhere^37^) to a) ensure that all participants had significant condition-related symptoms that affect health-related quality of life, such as breathlessness when hurrying (and exclusion if they achieved greater than 80% of their age-specific normative value on the International Shuttle Walk Test) b) to include ambulatory wheelchair users and c) to exclude people unsafe to exercise in a group without 1:1 or specialist supervision (e.g., people with significant risk of falls).

## Results

### Participant characteristics

During the intervention design process, we engaged with a diverse range of people living with MLTCs and their caregivers (n=26), as well as service provider staff (n=13), experts in multimorbidity/chronic illness (n=16) and commissioners (n=14). The characteristics of the stakeholders are shown in Tables 2 and 3. Participants had a wide range of age, gender and ethnicity (Table 2).

**Table 2.** Characteristics of the people from the lived experience advisory group.

Total N	26 (18 people with MLTCs, 8 caregivers)
Mean age (range)	61(42-82) years
Female (N (%))	16 (61.5%)
White British (N (%))	13 (50%)
British Indian (N (%))	5 (19%)
White other (N (%))	4 (15.5%)
Mixed Asian (N (%))	3 (11.5%)
Black African (N (%))	1 (4%)
LTCs reported	Attention deficit hyperactivity disorder, Angina, anxiety, arthritis (osteoarthritis and rheumatoid), Asthma, Atrial fibrillation, Autism, Back pain, Blindness, Bronchiectasis, cancer, chronic kidney disease, chronic pain, Cirrhosis of the liver, chronic obstructive pulmonary disease, coronary heart disease, Costochondritis, Dementia, depression, diabetes (type 1 and type 2), Ehlers-Danlos syndrome, Emphysema, Fibromyalgia, gastroparesis, glaucoma, hard of hearing, Hashimoto’s disease, heart failure, high cholesterol, high blood pressure, hypertrophic cardiomyopathy, Ileostomy, Incontinence (faecal and urinary), Insomnia, Intestinal failure, knee pain, Long-COVID, Lumbar spondylosis, Lupus, mechanical heart valve, migraine, mild cognitive impairment (early onset alzheimer’s), neuralgia, osteopenia, osteoporosis, pacemaker, partial paralysis, peripheral artery disease, pulmonary thromboembolic disease, renal artery stenosis, sinus tachycardia, Sjogren’s syndrome, sleep apnoea, sleep problems (periodic limb movement disorder), stoma, tendinitis/bursitis
Number of people with 2 LTCs (N (%))	3 (17%)
Number of people with 3 LTCs (N (%))	3 (17%)
Number of people with 4 LTCs (N (%))	4 (22%)
Number of people with 5 or more LTCs (N (%))	8 (44%)

**Table 3.** Characteristics of the service provider and commissioners groups and the PERFORM expert panel.

Service providers -professional roles (n=13)	Consultant chest physician, research physiotherapist, consultant physiotherapist and cardiovascular prevention and rehabilitation lead, lead exercise physiologist, senior pulmonary rehabilitation physiotherapist, community diabetes nurse, consultant renal physiotherapist, professor of healthcare research and lead for rehabilitation research group, professor of rehabilitation sciences, local NHS talking therapies LTCs lead, NIHR doctoral research fellow, NIHR senior clinical research fellow and honorary consultant geriatrician
Experts in multimorbidity/chronic illness (n=16)	Head of pulmonary and cardiac rehabilitation, chair of population health research, professor of general practice, chair of cardiovascular health, professor of psychology Applied to rehabilitation and health, clinical nurse manager for cardiac and pulmonary rehabilitation, clinical associate professor and honorary consultant respiratory physician, professor of health economics, professor of behavioural sciences and health, professor of public health and primary care, professor in LTCs research, clinical senior Lecturer/Honorary consultant, professor of psychology Applied to health, honorary clinical professor
Commissioners and policy group – professional roles or institutions (n=14)	Health education England, NHS England respiratory programme, professor of clinical Epidemiology, Chartered society of psychotherapy, richmond group of charities, NHS noncommunicable diseases for sustainability, scottish government national advisory Board for rehabilitation, professor of primary care, royal College of GPs, cardiovascular rehabilitation service lead and BACPR Council member

### Key messages from the stakeholder groups

The workshops with our key informant groups yielded unique insights into people with MLTCs’ and healthcare professionals’ needs and preferences, and the influences (including implementation context) that might impact the scale up of the intervention. The key messages from people with MLTCs and their supporters/Lived Experience Advisory Group included:

• *Unmet needs – a lack of guidance about how to exercise safely and how to manage the psychological consequences of having MLTCs.*
• *Main barriers to exercise include safety concerns and fear of pain or injury.*
• *A key concern about existing rehabilitation programmes was the lack of post-programme support.*
• *The importance of involving significant others during and after the programme, but without alienating those who lack positive sources of support.*
• *Accessibility of sessions (alternative models of delivery) and materials (e.g., text-only, Braille, different languages, video recordings of sessions, images capturing people from diverse ethnic populations) and locations (e.g., use of community-based as well as hospital locations)*.

The key messages from healthcare professionals were mostly focused on ensuring the safety of delivery and concerns about needing to become experts on numerous LTCs. The key messages included:

• *Ensuring the safety of people with MLTCs is paramount.*
• *Individual assessment and tailoring.*
• *Autonomy to collect data that are compliant with existing local audit/registration processes.*
• *Making the programme applicable to a wide audience (different ability groups) with a focus on common symptoms rather than specific LTCs.*
• *Not to ‘over-educate’ staff during the PERFORM training – healthcare professionals need to be permitted not to know everything and to use signposting for more complex issues/questions.*

Key messages from commissioners and policy makers focused largely on future implementation. These included:

• *Positioning of the new model within the UK healthcare system – hospital-based care vs primary/community-based. For example, possible commissioning through emerging Primary Care Networks or through an ‘additional roles’ reimbursement scheme.*
• *Using the wider workforce (e.g., charity sector, exercise instructors), building capacity within the community, developing optimal collaboration between the community and more specialised support.*
• *Increasing the chances for commissioning (securing payment) through consideration of the wider benefits above and beyond reducing hospital attendances/admissions (e.g., relieving pressure on general practice, reducing the need for social care, returning to (or staying in) work, reducing incidence of further LTCs)*.
• *Widening the target population – to introduce the intervention earlier in the person’s disease trajectory. For example, to offer the intervention shortly after diagnosis of the second LTC, or to people with cognitive impairment with only one LTC*.

### Behavioural specification

The workshop outputs and ongoing discussions with the PERFORM expert advisory group led to the development of a set of behavioural targets for people with MLTCs and for healthcare professionals. These are listed in Table 4.

**Table 4.** PERFORM intervention behavioural targets for people with MLTCs and healthcare professionals.

Behavioural targets (people with MLTCs)	Behavioural targets (healthcare professionals)
Engaging in supervised sessions of aerobic and resistance exercise to build or maintain exercise capacity (drawing on the approach used for existing pulmonary and cardiac rehabilitation services)	*Initial assessment:* Assessing individual needs and concerns (esp. About exercise) and identifying any requirements for exercise adaptation. Providing condition-specific information where needed. Using a mixed communication style (asking both closed and open questions to elicit information, but more open questions, reflective listening/ask-tell-ask to discuss any barriers and improve understanding). Assessing exercise capacity using an appropriate test
Increasing engagement with home-based/day-to-day physical activity both during and after the PERFORM programme	*Group-based exercise sessions:* Providing an individually tailored exercise prescription, based initially on the individual’s exercise capacity. Ensuring safe/correct performance of exercises. Monitoring progress and progressing/regressing exercise intensity or effort to achieve moderate intensity. Encouraging self-monitoring and adjustment of effort using a 0-10 effort scale. Using a mixed communication style (as above)
Engaging in self-management behaviours related to managing pain, managing stress, managing low mood, managing breathlessness, managing fatigue, eating healthily, sleeping well and managing medication. This includes engaging in (both attending and participating in) the PERFORM health and wellbeing sessions	*Home-based exercise prescription:* Agreeing on an individually tailored home-based exercise programme for each person. Monitoring progress (using the PERFORM progress tracker workbook) and progressing/regressing exercise intensity or effort to achieve moderate intensity. Using a mixed communication style (as above)
	*Health and Wellbeing sessions (including maintenance sessions):* Using a person-centred communication style (open questions, ask-tell-ask, reflective listening) to deliver all the intended educational content and interactive exercises in each session. Encouraging and supporting action-planning. Reviewing progress with planned changes in self-management behaviours. Supporting and encouraging problem-solving to overcome any barriers in changing self-management behaviours. Acknowledging/normalising the psychological consequences of living with MLTCs and helping the person with MLTCs find ways to address them
	*Discharge appointment:* Using a person-centred communication style (as above). Reviewing progress made since the start of the programme. Discussing/agreeing plans for ongoing exercise and/or day-to-day physical activity and other key self-management behaviours. Identifying and trying to address barriers to ongoing exercise and key self-management behaviours. Identifying and exploring any remaining concerns or queries about their ongoing self-management. Assessing exercise capacity using the same exercise capacity test as for initial assessment
	*Caregiver involvement:* Encouraging involvement of any friends or family who are (and who are not) present during any of the sessions in supporting the intended self-management behaviours

Through the stakeholder workshops, we identified processes that might facilitate or hinder engagement in the target behaviours. These mediating processes are outlined in Supplemental Table S1 and S2 in Appendices 4 and 5. For example, for people with MLTCs, this might include increasing understanding of how lifestyle behaviours such as activity and diet can affect quality of life, improving the person’s social support network, developing motivation and self-efficacy for various self-management behaviours, and learning how to manage low mood, anxiety or stress. We then created a programme specification with strategies to mitigate the identified barriers and promote/activate the identified enabling factors.

### Programme theory

**Figure 1.**
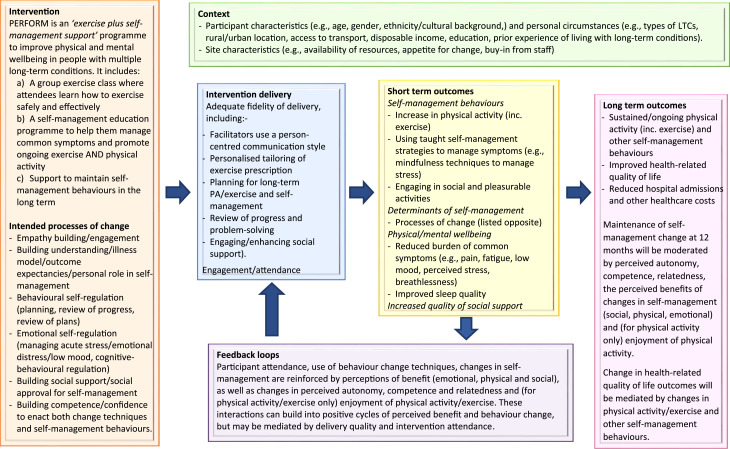
Logic model for the PERFORM intervention.

The theoretical and interventional processes intended to promote change in the target behaviours and the consequent changes in outcomes are summarised in the intervention logic model (Figure 1). The theoretical underpinnings/key principles of the PERFORM programme include:

- Building autonomy and empathy (using person-centred counselling).^38^
- Tailoring exercise and self-management support to individual needs.
- Self-regulation (building self-efficacy/competence via a cycle of realistic action-planning, self-monitoring, reviewing progress and problem-solving).^39^
- Building a functional ‘illness model’ (as defined in Leventhal’s Common Sense Model of self-regulation), to help people with MLTCs see how self-management behaviours affect their physical and mental wellbeing.^40^
- Facilitating the process of psychological adaptation to living with LTCs (via acceptance-based approaches/finding a new normal).^41^
- Engaging/building social support.^42^
- Cognitive behavioural strategies and mindfulness-based approaches to manage the psychological consequences of living with MLTCs.^43,44^

### Programme specification

**Figure 2.**
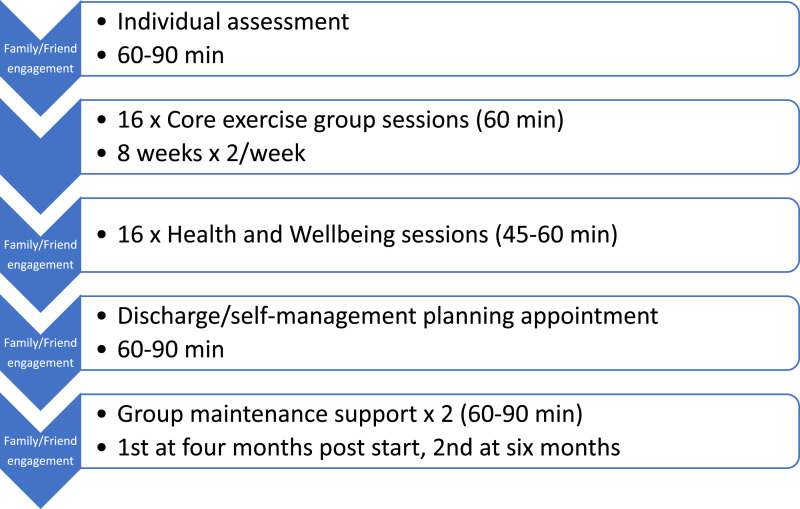
Structure of the PERFORM programme.

The PERFORM programme consists of several components that are sequenced as shown in Figure 2. These include a core set of 16 group exercise sessions, followed (or preceded) by 16 self-management-support (‘Health and Wellbeing’) sessions, delivered twice-weekly over eight weeks. To support maintenance of behaviour changes, two group follow-up sessions are delivered two and four months after the end of the initial eight-week programme. The programme was designed to be delivered by existing rehabilitation service staff such as specialist nurses, physiotherapists and exercise physiologists, with access to multidisciplinary team support (from condition-specific consultants, specialist nurses and a clinical psychologist or counsellor). Access to support from a pharmacist and a dietitian were also considered to be desirable.

All programme delivery details, for example, staff to patient ratio, must be consistent with recommendations for delivery of exercise-based rehabilitation programmes from the British Association for Cardiovascular Prevention and Rehabilitation Standards and Core Components for cardiovascular disease prevention and rehabilitation^34^ and the British Thoracic Society guideline on pulmonary rehabilitation in adults.^35^ Some delivery details will depend on different implementation contexts, for example, the team’s availability of space and staff, and therefore, group sizes can be adjusted accordingly. However, we recommended a maximum group size of 15 participants, and due to the interactive nature of the programme, a minimum group size of 6-8 participants. The programme can be delivered as a rolling programme (with participants joining and exiting at different times) or as a cohort programme (with all participants starting and finishing at the same time), depending on existing delivery modes, staff capacity and teams’ preferences.

All intervention materials were reviewed by the people with MLTCs and healthcare professional advisory groups, as well as by several members of the PERFORM research programme’s PPIE group (a separate advisory group of people with MLTCs convened to support the overall PERFORM research programme).

### Initial assessment

On receiving a referral for the PERFORM programme, the rehabilitation provider organises an individual assessment of the person with MLTCs to ascertain eligibility (Table 1), identify and address any specific needs, problematic/limiting symptoms or concerns and inform an initial exercise prescription (in terms of exercise modalities and intensity). The initial assessment also checks for any safety issues or specific exercise advice needed relating to the person’s LTCs. The assessment lasts up to 90 minutes and includes measurement and consideration of the person’s physical capabilities or limitations, their exercise capacity and any cautions or contraindications for specific types of exercise related to their LTCs. The initial assessment should also identify key symptoms of LTCs that may be modified by the PERFORM programme, perceived limitations to day-to-day activities, psychological status (with a particular focus on stress, emotional distress and low mood), availability of social support and goals that people with MLTCs want to work towards whilst attending the programme. Following the initial assessment, each person’s individual exercise prescription is tailored to their aerobic capacity and any limitations or exercise-related considerations linked with their LTCs.

At the end of their initial assessment, people with MLTCs receive several PERFORM handouts/resources. This includes information sheets that outline any disease-specific considerations for exercising (e.g., how to manage claudication pain in people with peripheral vascular disease). Additionally, people with MLTCs are asked to record their progress in a ‘Progress Tracker’ diary/workbook. This starts with their initial goals set at the beginning of treatment, physical and emotional wellbeing during the programme, levels of physical activity outside of the PERFORM sessions (e.g., walking, strength training or chair-based exercises), what they have learnt (and what self-management strategies they propose to try) in each Health and Wellbeing session and any longer-term goals they would like to work towards at the end of the programme.

Following the initial assessment, people with MLTCs are invited to attend twice-a-week rehabilitation sessions for eight weeks, each attendance consisting of one hour of group-based exercise (individually prescribed and progressed) - ‘Move and Improve’ sessions, and 45-60 minutes of self-management support - ‘Health and Wellbeing’ sessions. If possible, we recommend that the gap between the Initial Assessment and the start of the programme should not exceed four weeks. During the first ‘Move and Improve’ session, each person receives the PERFORM Home Exercise Booklet containing a set of exercises that are easy to perform at home (including individually prescribed aerobic walking and strength training) and which are individually tailored to the person’s needs, capacities and circumstances. Each person will be recommended to exercise three times per week (including their ‘Move and Improve’ sessions).

### ‘Move and Improve’ sessions

The PERFORM ‘Move and Improve’ exercise sessions aim to increase physical fitness, strength and balance, and efficiency of movement. The sessions are delivered twice per week. They follow the format of existing cardiac/pulmonary rehabilitation programmes that are widely delivered in the NHS. They also follow the principles of existing guidelines for such programmes as outlined by the British Association for Cardiovascular Prevention and Rehabilitation^34^ and the British Thoracic Society guideline on pulmonary rehabilitation in adults.^35^ Each session consists of a warm-up at the beginning and a cool-down at the end. Participants undertake an individually prescribed and progressed aerobic and resistance exercise programme working all major muscle groups. For example, this may include stationary bikes, treadmills, and (functionally oriented) resistance exercises using resistance bands and/or weights, and isometric exercises (depending on the venue and availability of equipment). Exercise ‘stations’ are offered, tailored to each person’s individual needs and limitations (e.g., joint pain, poor balance, low muscle strength). Chair-based exercises are offered to people who struggle with standing exercise.

Rehabilitation staff monitor attendees to make sure they are safe whilst exercising with the aim of working at a moderate intensity (i.e., 60-70% of their exercise capacity (for example, calculated from an Incremental Shuttle walk Test^45^)). Standard rehabilitation-service practices for ensuring readiness to exercise are used prior to and during each session, including symptom-checking and monitoring of exercise intensity and quality/safety of movement. Certain LTCs may require additional monitoring before, during and after each exercise session (for example, monitoring glucose levels for people with insulin-dependent diabetes). PERFORM facilitators use a 0-10 effort scale to anchor the person’s understanding of how it feels to work at moderate intensity (between levels 4-7 on the scale).^46,47^ The PERFORM facilitators aim to progress in each session the difficulty and intensity of exercises within each participant’s capabilities to maximise the benefits in terms of aerobic fitness, functional muscle strength and balance. The initial exercise prescription is reviewed (and if necessary, adjusted) after the first exercise session depending on the person’s response, including their effort rating and how they feel the next day.

Participants are advised to exercise at home on at least one further day using a home-based exercise prescription (using an individually tailored set of resistance exercises using weights found around the home (e.g., filled water bottles or tins) with a 5-minute warm-up and cool-down process). The PERFORM facilitators adapt (progress or regress) the home-based exercise prescription based on the progress made (as recorded in the person’s Progress Tracker and via discussion within the exercise session) and any recent changes in their physical status (e.g., recovering from a cold or absence from exercising).

### ‘Health and Wellbeing’ sessions

**Figure 3.**
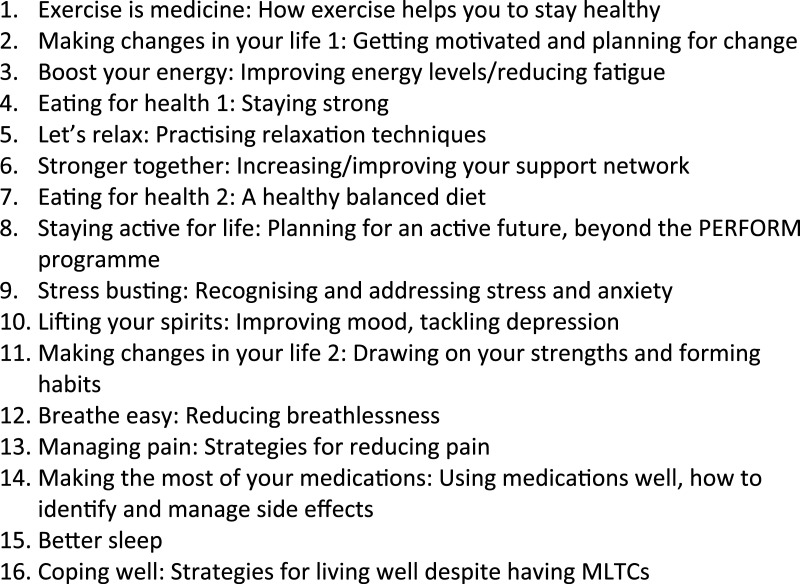
Health and Wellbeing session topics.

The ‘Health and Wellbeing’ sessions are semi-structured self-management support sessions designed to help people with MLTCs to improve their physical and mental health and wellbeing. The sessions cover 16 topics that are pertinent to people living with MLTCs (these can be delivered in any order, so it can be delivered as a ‘rolling’ programme, with people joining at any point in the cycle). Most topics relate to a common physical symptom (e.g., fatigue, pain, breathlessness) or psychological consequence associated with living with MLTCs. Five of the sessions relate to more generic self-management support processes (e.g., engaging social support, managing medications, planning and making changes in your life). Each session presents evidence-based information and supports the interactive discussion of evidence-based self-management strategies. The session topics are listed in Figure 3.

The sessions are structured around PowerPoint presentations and use a person-centred communication style (e.g., asking open-ended questions, using reflective listening)^48,49^ with an overarching ‘ask-tell-ask’ format to: (1) ascertain the group’s knowledge on the topic (ask – for example, ‘what things can you do to improve your mood?’); (2) introduce effective self-management techniques (tell – for example, physical activity and talking therapies can help with low mood) and (3) invite group discussion to foster behavioural change (discuss – for example, what is the learning you are taking away from today’s session? what are you going to do differently?).

Making a commitment to long-term behavioural change, both in terms of increasing physical activity as well as using self-management strategies, is an important component of the ‘Health and Wellbeing’ sessions. Hence, towards the end of each session, people are encouraged to record their learning in their PERFORM Progress Trackers, this can include recording the main take-away messages or planning to test out the suggested self-management strategies. People with MLTCs also receive a set of printed handouts consolidating the covered topic, as well as facilitating additional learning (e.g., by recommending further resources or signposting to organisations or charities specialising in the subject matter). Water is available and attendees are encouraged to drink throughout the exercise sessions and post exercise (during the Health and Wellbeing sessions). If a person misses a session, they can access the learning materials and a planning worksheet from a website (during the PERFORM trial, this will only be available to intervention group participants).

### End-of-core-programme/self-management planning appointment

At the end of the exercise programme (after eight weeks), each person is invited to an individual self-management planning appointment. During this meeting, people can review the progress they have made whilst attending the PERFORM programme, as well as make a long-term plan about how they can sustain engagement in exercise and/or more general physical activity and other individually relevant self-management behaviours.

### Additional follow-up/maintenance support sessions

During the above appointment the PERFORM facilitator invites each person to two follow up sessions – two months and four months after they finished the programme. These group-based maintenance-support sessions provide an opportunity for attendees to review their long-term progress, to identify and address any barriers to change (or maintenance of change) that they have encountered and answer any further questions they may have about engaging in physical activity and using the self-management strategies introduced during the programme.

### Engaging social support

To maximise the impact and longevity of the intervention, attendees are encouraged to involve a trusted family member or a friend during the different stages of the programme. For example, they may be able to contribute to the assessment process or attend the maintenance sessions to offer support when the programme comes to an end. One of the self-management support sessions (‘Stronger together’) is also focused on engaging social support to constructively help people with MLTCs to plan and implement their self-management support strategies. During this session, attendees are encouraged to consider three theoretical functions of social support: informational support, emotional support and practical support.^42^

### Facilitator training programme

A training manual and syllabus for a 1-day training course for PERFORM facilitators was developed. The facilitation role is crucial to the success of the PERFORM programme. As well as being the main delivery vehicle, it enables tailoring of intervention resources to the individual needs of people with MLTCs. The course includes the process of facilitation (building rapport using person-centred counselling techniques, empowerment and support of self-management); delivery of the exercise programme; techniques for managing stress, emotional distress and low mood; contents and delivery of the Health and Wellbeing sessions; supporting exercise and physical activity using the intervention materials. The training includes opportunities to practice facilitation techniques and to discuss any concerns or issues around local implementation.

### Refinement

Feedback from a randomised mixed methods feasibility trial (to be published elsewhere) was used to refine the intervention (the final version being as described above). Refinements made at this stage included the following:

- Restructuring the method of presentation for the Health and Wellbeing sessions to structurally embed intended behaviour change techniques (action-planning, reviewing of progress, using the ‘ask-tell-ask’ approach).
- Additional training in delivery of key, intended behaviour change techniques (including action-planning, reviewing of progress and engaging social support)
- Extending the number of Health and Wellbeing sessions from 14 to 16 (2 sessions per week for 8 weeks).
- Increasing flexibility so that participants for whom the topic of the session was not as relevant could still engage in activity relevant to their own self-management.
- More individual-tailoring opportunities and small group work.
- Clearer information about the maintenance sessions, what they will involve and reminders.
- Including content to emphasise the usefulness of action-planning.
- Providing clearer and more accessible schedule options (e.g., later start time) and ensuring opportunities to make up missed sessions (we created a website for patients and providers to access all patient-facing materials).
- Making the Health and Wellbeing sessions more interactive.
- Making the Progress Tracker easier to use.
- Creating a recorded version of the facilitator training and video-examples of intended delivery for two of the Health & Wellbeing sessions.
- Changes to the eligibility criteria to ensure that participants experience significant condition-related symptoms.

## Discussion

This paper describes the development of the PERFORM intervention for people with MLTCs.

Few other studies have described in detail the development, theoretical basis, intervention techniques and strategies for an intervention for promoting the quality of life of people with MLTCs. The MOBILIZE intervention is an exercise therapy and self-management support for people with multimorbidity that is currently undergoing evaluation in Denmark.^50^ Like PERFORM, this has been designed to work in both hospital and community settings and was co-developed with people with MLTCs, caregivers and care providers, although it is delivered by physiotherapists only. It consists of twice-weekly exercise (60 minutes) followed by 30-minute self-management sessions for 12-weeks. In common with PERFORM, the self-management sessions target key self-management skills/topic, including mindfulness, breathing exercises, managing mental health symptoms, healthy eating and managing pain and maintenance of exercise and self-management beyond the 12-week programme. The sessions use experiential exercises, group discussions and homework to engage participants in action planning, problem-solving and engaging social support around these self-management targets. The main difference between the two interventions is the longer duration of the MOBILIZE programme, as well as the difference in populations (MOBILIZE targets a narrower range of conditions) and healthcare contexts (Denmark versus the UK).^9^ The forthcoming PERFORM and MOBILIZE randomised trials (both of which have health-related quality of life (EQ-5D) as the primary outcome) will reveal how these two comprehensive MLTCs interventions perform in practice.

### Strengths and limitations

The Person Based Approach^26^ provided a clear structure and process for the co-creation of the intervention. The process accounted for the needs of a diverse range of stakeholders including people with MLTCs, their caregivers, healthcare professionals, potential facilitators and healthcare commissioners. As such, the intervention is grounded in evidence, theory and best practice, as well as the perspectives of people with MLTCs, healthcare professionals and commissioners. The construction of a programme theory and causal logic model (Figure 1), as recommended by the Medical Research Council framework on complex intervention development, provided a useful framework for integrating the targets for change, the processes/mechanisms designed to effect these changes and contextual factors influencing the impact of the intervention.^25^

Despite a transparent audit trail and documentation of all the processes involved, it must be acknowledged that a different team and set of stakeholders might have developed a very different intervention with a different delivery format and a different theoretical basis. Judgement calls on a range of pragmatic and theoretical choices were required at many stages. Although our multi-stakeholder approach ensured that decisions were based on either evidence, existing best practice or appropriate stakeholder expertise, there was often no clear ‘best solution’. Hence, as it has been previously noted, intervention development remains as much an art as a science,^46^ depending largely on the expertise, experience, instincts and knowledge of the intervention development team and the stakeholder groups involved and the quality of their interactions. Despite this potential for variation, it is interesting and encouraging to note the consistency of our approach with the MOBILIZE intervention described above.

### Implications and future directions

Further research is now needed to assess the clinical effectiveness and cost-effectiveness of the PERFORM intervention. A planned multi-site, fully powered randomised trial will include a mixed methods process evaluation to assess delivery fidelity, implementation and mechanisms of change specified in our intervention programme theory and logic model (Figure 1) which will lead to further refinement of the intervention to maximise potential for future wide scale implementation.

## Conclusions

Using intensive and diverse stakeholder involvement we have developed a comprehensive, evidence-informed, theoretically driven, facilitated self-management and rehabilitation intervention, that is grounded in the needs of people with MLTCs (and their supporters), service providers and service commissioners, which is now ready for a large-scale evaluation.

## Supplemental Material

Supplemental Material - Co-design and development of a personalised exercise-based rehabilitation and self-management programme for people with multiple long-term conditions: The PERFORM intervention Supplemental Material for Co-design and development of a personalised exercise-based rehabilitation and self-management programme for people with multiple long-term conditions: The PERFORM intervention by Paulina Daw, Colin J. Greaves, Nikki Gardiner, Patrick Doherty, Thomas M. Withers, Amy C. Barradell, Paul O’Halloran, Zahira Ahmed, Shaun Barber, Gwen Barwell, Sophie E. Brown, Sarah Dean, Carlos Echevarria, Rachael A. Evans, Tracy Ibbotson, Bhautesh D. Jani, Kate Jolly, James R. Manifield, Frances S. Mair, Emma McIntosh, Dan Miller, Paula Ormandy, Susan M. Smith, Sharon A. Simpson, Ghazala Waheed, Rod S. Taylor, Sally J. Singh and on behalf of PERFORM research team in Journal of Multimorbidity and Comorbidity

Supplemental Material - Co-design and development of a personalised exercise-based rehabilitation and self-management programme for people with multiple long-term conditions: The PERFORM intervention Supplemental Material for Co-design and development of a personalised exercise-based rehabilitation and self-management programme for people with multiple long-term conditions: The PERFORM intervention by Paulina Daw, Colin J. Greaves, Nikki Gardiner, Patrick Doherty, Thomas M. Withers, Amy C. Barradell, Paul O’Halloran, Zahira Ahmed, Shaun Barber, Gwen Barwell, Sophie E. Brown, Sarah Dean, Carlos Echevarria, Rachael A. Evans, Tracy Ibbotson, Bhautesh D. Jani, Kate Jolly, James R. Manifield, Frances S. Mair, Emma McIntosh, Dan Miller, Paula Ormandy, Susan M. Smith, Sharon A. Simpson, Ghazala Waheed, Rod S. Taylor, Sally J. Singh and on behalf of PERFORM research team in Journal of Multimorbidity and Comorbidity

Supplemental Material - Co-design and development of a personalised exercise-based rehabilitation and self-management programme for people with multiple long-term conditions: The PERFORM intervention Supplemental Material for Co-design and development of a personalised exercise-based rehabilitation and self-management programme for people with multiple long-term conditions: The PERFORM intervention by Paulina Daw, Colin J. Greaves, Nikki Gardiner, Patrick Doherty, Thomas M. Withers, Amy C. Barradell, Paul O’Halloran, Zahira Ahmed, Shaun Barber, Gwen Barwell, Sophie E. Brown, Sarah Dean, Carlos Echevarria, Rachael A. Evans, Tracy Ibbotson, Bhautesh D. Jani, Kate Jolly, James R. Manifield, Frances S. Mair, Emma McIntosh, Dan Miller, Paula Ormandy, Susan M. Smith, Sharon A. Simpson, Ghazala Waheed, Rod S. Taylor, Sally J. Singh and on behalf of PERFORM research team in Journal of Multimorbidity and Comorbidity

Supplemental Material - Co-design and development of a personalised exercise-based rehabilitation and self-management programme for people with multiple long-term conditions: The PERFORM intervention Supplemental Material for Co-design and development of a personalised exercise-based rehabilitation and self-management programme for people with multiple long-term conditions: The PERFORM intervention by Paulina Daw, Colin J. Greaves, Nikki Gardiner, Patrick Doherty, Thomas M. Withers, Amy C. Barradell, Paul O’Halloran, Zahira Ahmed, Shaun Barber, Gwen Barwell, Sophie E. Brown, Sarah Dean, Carlos Echevarria, Rachael A. Evans, Tracy Ibbotson, Bhautesh D. Jani, Kate Jolly, James R. Manifield, Frances S. Mair, Emma McIntosh, Dan Miller, Paula Ormandy, Susan M. Smith, Sharon A. Simpson, Ghazala Waheed, Rod S. Taylor, Sally J. Singh and on behalf of PERFORM research team in Journal of Multimorbidity and Comorbidity

Supplemental Material - Co-design and development of a personalised exercise-based rehabilitation and self-management programme for people with multiple long-term conditions: The PERFORM intervention Supplemental Material for Co-design and development of a personalised exercise-based rehabilitation and self-management programme for people with multiple long-term conditions: The PERFORM intervention by Paulina Daw, Colin J. Greaves, Nikki Gardiner, Patrick Doherty, Thomas M. Withers, Amy C. Barradell, Paul O’Halloran, Zahira Ahmed, Shaun Barber, Gwen Barwell, Sophie E. Brown, Sarah Dean, Carlos Echevarria, Rachael A. Evans, Tracy Ibbotson, Bhautesh D. Jani, Kate Jolly, James R. Manifield, Frances S. Mair, Emma McIntosh, Dan Miller, Paula Ormandy, Susan M. Smith, Sharon A. Simpson, Ghazala Waheed, Rod S. Taylor, Sally J. Singh and on behalf of PERFORM research team in Journal of Multimorbidity and Comorbidity

## Data Availability

The data that support the findings of this study are available from the corresponding author, [PD], upon reasonable request. *

## ORCID iDs

Paulina Daw https://orcid.org/0000-0002-0942-3953

Thomas M. Withers https://orcid.org/0000-0002-5286-7189

Paul O’Halloran https://orcid.org/0000-0001-6495-2359

Shaun Barber https://orcid.org/0000-0002-8073-2687

Gwen Barwell https://orcid.org/0009-0002-8500-175X

Rachael A. Evans https://orcid.org/0000-0002-1667-868X

Tracy Ibbotson https://orcid.org/0000-0002-9224-6467

Bhautesh D. Jani https://orcid.org/0000-0001-7348-514X

Frances S. Mair https://orcid.org/0000-0001-9780-1135

Susan M. Smith https://orcid.org/0000-0001-6027-2727

Sharon A. Simpson https://orcid.org/0000-0002-6219-1768

Ghazala Waheed https://orcid.org/0000-0002-5142-110X
