# Supplementary material for: Co-design and development of a Personalised Exercise-based Rehabilitation and self-management programme FOR people with Multiple long-term conditions: The PERFORM intervention
Source: J Multimorb Comorb. 2025 Sep 18;15:26335565251367326. doi: 10.1177/26335565251367326 (PMC12446826; doi:10.1177/26335565251367326)
Supplement: Supplemental Material - Co-design and development of a personalised exercise-based rehabilitation and self-management programme for people with multiple long-term conditions: The PERFORM intervention [file sj-pdf-2-cob-10.1177_26335565251367326.pdf]

TIDieR
Template for Intervention Description and Replication

The TIDieR (Template for Intervention Description and Replication) Checklist*:

Information to include when describing an intervention and the location of the information

Item number	Item	Where located ** Primary paper (page or appendix number)	Other † (details)
	**BRIEF NAME**		
**1.**	Provide the name or a phrase that describes the intervention.	The last paragraph of the Background section	______
	**WHY**		
**2.**	Describe any rationale, theory, or goal of the elements essential to the intervention.	The last paragraph of the Background section, the last paragraph of the Methods section, 'Theoretical underpinnings and logic model' section within Results, the first paragraph of the 'Programme specification' section within Results and the last paragraph of the 'Health and Wellbeing sessions' section within Results	______
	**WHAT**		
**3.**	Materials: Describe any physical or informational materials used in the intervention, including those provided to participants or used in intervention delivery or in training of intervention providers. Provide information on where the materials can be accessed (e.g. online appendix, URL).	The second and third paragraphs of the 'Initial assessment' section within Results, third paragraph of the 'Health and Wellbeing sessions' section within Results and 'Training programme' section within Results	______
**4.**	Procedures: Describe each of the procedures, activities, and/or processes used in the intervention, including any enabling or support activities.	The following sections within Results:<br>- 'Initial assessment'<br>- 'Move and Improve sessions'<br>- 'Health and Wellbeing' sessions<br>- 'End-of-core-programme / self-management planning	______

		appointment’<br>- ‘Additional follow-up/maintenance support sessions’	
	**WHO PROVIDED**		
**5.**	For each category of intervention provider (e.g. psychologist, nursing assistant), describe their expertise, background and any specific training given.	End of the first paragraph of the ‘Programme specification’ section within Results and ‘Training programme’ section with Results	______________
	**HOW**		
**6.**	Describe the modes of delivery (e.g. face-to-face or by some other mechanism, such as internet or telephone) of the intervention and whether it was provided individually or in a group.	Figure 2	______________
	**WHERE**		
**7.**	Describe the type(s) of location(s) where the intervention occurred, including any necessary infrastructure or relevant features.	‘Results’ section in the Abstract and “Programme Specification” section in Results.	______________
	**WHEN and HOW MUCH**		
**8.**	Describe the number of times the intervention was delivered and over what period of time including the number of sessions, their schedule, and their duration, intensity or dose.	Figure 2	______________
	**TAILORING**		
**9.**	If the intervention was planned to be personalised, titrated or adapted, then describe what, why, when, and how.	The first and third paragraphs of the ‘Initial assessment’ section within Results, the first and third paragraphs of the ‘Move and Improve sessions’ section within Results	______________
	**MODIFICATIONS**		
**10.**[ǂ]	If the intervention was modified during the course of the study, describe the changes (what, why, when, and how).	Intervention development is an iterative process, so this is covered across the manuscript, as well as specifically (for actioning feedback	

		from our feasibility study) in the 'Refinements' section at the end of the Results section.
	**HOW WELL**	
**11.**	Planned: If intervention adherence or fidelity was assessed, describe how and by whom, and if any strategies were used to maintain or improve fidelity, describe them.	Intervention fidelity data will be reported elsewhere /within the PERFORM Feasibility Study report
**12.**ǂ	Actual: If intervention adherence or fidelity was assessed, describe the extent to which the intervention was delivered as planned.	

** **Authors** - use N/A if an item is not applicable for the intervention being described. **Reviewers** – use '?' if information about the element is not reported/not sufficiently reported.

† If the information is not provided in the primary paper, give details of where this information is available. This may include locations such as a published protocol or other published papers (provide citation details) or a website (provide the URL).

ǂ If completing the TIDieR checklist for a protocol, these items are not relevant to the protocol and cannot be described until the study is complete.

* We strongly recommend using this checklist in conjunction with the TIDieR guide (see *BMJ* 2014;348:g1687) which contains an explanation and elaboration for each item.

* The focus of TIDieR is on reporting details of the intervention elements (and where relevant, comparison elements) of a study. Other elements and methodological features of studies are covered by other reporting statements and checklists and have not been duplicated as part of the TIDieR checklist. When a **randomised trial** is being reported, the TIDieR checklist should be used in conjunction with the CONSORT statement (see www.consort-statement.org) as an extension of **Item 5 of the CONSORT 2010 Statement.** When a **clinical trial protocol** is being reported, the TIDieR checklist should be used in conjunction with the SPIRIT statement as an extension of **Item 11 of the SPIRIT 2013 Statement** (see www.spirit-statement.org). For alternate study designs, TIDieR can be used in conjunction with the appropriate checklist for that study design (see www.equator-network.org).
