# Supplementary material for: Co-design and development of a Personalised Exercise-based Rehabilitation and self-management programme FOR people with Multiple long-term conditions: The PERFORM intervention
Source: J Multimorb Comorb. 2025 Sep 18;15:26335565251367326. doi: 10.1177/26335565251367326 (PMC12446826; doi:10.1177/26335565251367326)
Supplement: Supplemental Material - Co-design and development of a personalised exercise-based rehabilitation and self-management programme for people with multiple long-term conditions: The PERFORM intervention [file sj-pdf-3-cob-10.1177_26335565251367326.pdf]

Appendix 3

Appendix 3: Group workshop aims and topics

Aims	Topics explored	Stakeholder input		
		Patients and carers	Healthcare professionals	Commissioner and policy makers
Identification of common symptoms associated with living with multiple long-term conditions	What symptoms are commonly associated with living with multiple long-term conditions?	x	x	
Identification of patients' and carers' needs	What are the needs and concerns of people living with multiple long-term conditions?	x	x	
Identification of service delivery gaps	What support is missing in the NHS to support patients living with multiple long-term conditions?	x	x	x
	What are the challenges of providing rehabilitation to patients with multiple long-term conditions?		x	x
	What support is already available for those with multiple long-term conditions who attend your rehab services/how do you currently manage or adapt your service for multiple long-term conditions? What support is missing?		x	
Identification of barriers and enablers to exercise	What gets in the way of exercising (barriers) and what helps people to exercise (supporting influences)?	x		

	What might (1) get in the way of or (2) help people to exercise at home – in-between weekly rehabilitation sessions?<br><br>What might (1) get in the way of or (2) help people to continue exercising after a (typically 8-12 week) exercise-based rehabilitation programme?<br><br>How best to encourage /facilitate the between-sessions (home-based) exercise prescription?	x<br><br><br>x<br><br><br>x	 <br><br><br><br><br>x	
Identification of barriers and enablers to self-management behaviour change	What will make participants actually do /implement the strategies introduced during PERFORM (e.g. stress-management, engaging social support)? What might get in the way?<br><br>Format of self-management sessions (e.g., group-based post-exercise, online, other)	x<br><br><br><br>x	 <br><br><br>x	
Identification of topics that need to be included during the self-management sessions	Which topics should be explored during the self-management sessions?<br><br>What should be the content of the PERFORM materials?	x<br><br>x	x<br><br>x	
Identification of the content of the initial assessment and end-of-programme conversation/meeting	What questions need to be asked /what tests need to be performed during the initial assessment meeting?<br><br>What questions need to be asked /tests need to be performed during the end-of-programme meeting?	x<br><br><br>x	x<br><br><br>x	
Identification of training needs	What do we need to include during the PERFORM training for healthcare professionals, so they can deliver the intervention well?<br><br>What support or training is already available for healthcare professionals treating patients with multiple long-term conditions?	x	x<br><br><br>x	
Identification of implementation barriers and enablers	Are there any particular considerations when exercising multimorbid patients and when providing patient education?		x	

	How would you feel if you were required to offer treatment to a wider set of patients?		x	
	What would help you to adjust to this new way of working?		x	
	What kind of programme would you ideally like to deliver for these patients?		x	
	What kind of programme would be realistic to deliver bearing in mind current resources?		x	x
	What would be the challenges of implementing this new multimorbidity model?			x
	Assuming the intervention is effective and cost-effective: How can it fit within the NHS? What other evidence or information would help with commissioning/implementation? What else might make it easier to implement? Who would you need to involve to set up a system like this?			x
	The bigger picture/system level change: Are there any additional data/outputs we could provide, for example, a guide for practitioners/a guide for commissioners? What information are the commissioners likely to need, for example, a commissioning brief?			x
