# Supplementary material for: Co-design and development of a Personalised Exercise-based Rehabilitation and self-management programme FOR people with Multiple long-term conditions: The PERFORM intervention
Source: J Multimorb Comorb. 2025 Sep 18;15:26335565251367326. doi: 10.1177/26335565251367326 (PMC12446826; doi:10.1177/26335565251367326)
Supplement: Supplemental Material - Co-design and development of a personalised exercise-based rehabilitation and self-management programme for people with multiple long-term conditions: The PERFORM intervention [file sj-pdf-4-cob-10.1177_26335565251367326.pdf]

Supplementary Table S1: Behavioural targets, associated barriers and enablers, theoretical processes of change and strategies to effect change for patients in the PERFORM intervention.

Key behavioural targets	Modifiable barriers /enablers	Theoretical processes* (barriers addressed)	Behaviour change techniques* (processes addressed)	Specific strategies/intervention techniques (examples)
Engaging in physical activity during the PERFORM programme<br><br>This includes:<br><br>Attending the PERFORM supervised exercise sessions	**Individual barriers**<br>1. Experiencing pain/discomfort during or after exercise<br>2. Safety concerns – fear of making something worse (e.g. joint pain, heart condition)<br>3. Exercise is not a priority (caring/parental responsibilities, employment responsibilities, etc.)<br>4. Not knowing where to start or which exercises will be right<br>5. Friends and family can discourage engagement<br><br>**Individual enablers**<br>6. Understanding the physical, mental and other benefits associated with exercise /PA<br>7. Experience of working at the right/moderate intensity (pacing, not doing too much, or too little)<br>8. Learning that exercise can be safe<br>9. Becoming more confident in engaging in exercise over time<br>10. Experiencing or measuring the impact of (benefits) and progress made during the programme<br>11. Making exercise sessions enjoyable, for example, by playing music, playing games<br>12. Group support within sessions<br>13. Involving the patient's wider support network<br>14. Peers recognise/acknowledge the benefits of PA and/or exercise	a. Knowledge: Awareness of the existence of something (4, 6, 8)<br><br>b. Skill: An ability or proficiency acquired through practice (7, 9)<br><br>c. Beliefs about capabilities: Beliefs about one's ability to successfully carry out a behaviour (2, 9)<br><br>d. Social influences: Interpersonal processes that can influence one's thoughts, feelings or behaviours (5, 12, 13,14)<br><br>e. Motivation: The impetus that gives purpose or direction to behaviour and operates at a conscious or unconscious level (1, 2, 3, 11)<br><br>f. Emotion: A complex reaction pattern involving	Goal setting (e,k)<br><br>Problem solving (c,b,h)<br><br>Action planning (e)<br><br>Feedback on behaviour (and ideas) (a,c,e,g)<br><br>Self-monitoring of behaviour (g)<br><br>Self-monitoring of outcomes of behaviour (f,j)<br><br>Individual tailoring of advice and support (not in BCW or taxonomy) (e,j)<br><br>Review behaviour goals (k)	Goal setting and review throughout the programme: initial assessment, exercise sessions, Health and Wellbeing sessions, discharge appointment and maintenance sessions<br><br>Action-plans with embedded problem solving (coping plan) and social support planning – in both handouts and sessions<br><br>Problem-solving and review of progress within sessions<br><br>Using a Progress Tracker booklet to self-monitor progress with exercise and self-care and link this to changes in physical and mental wellbeing.

Key behavioural targets	Modifiable barriers /enablers	Theoretical processes* (barriers addressed)	Behaviour change techniques* (processes addressed)	Specific strategies/intervention techniques (examples)
	**System-level barriers**<br>15. Conflicting advice from different healthcare professionals /a lack of buy-in from healthcare professionals for encouraging physical activity<br>16. Overreliance on medical procedures and medication (both by healthcare professionals and patients /carers)<br>17. Payment for/only part-funded programmes<br><br>**System-level enablers**<br>18. Low cost (free at the point of access)<br>19. Programme availability – inclusive catchment areas, no /short waiting times, ease of access .travel<br>20. Diversity of delivery - choice of modes, different venues (home-based, virtual, hybrid), translations (e.g. Braille)<br>21. Access to specialised advice on what is safe<br>22. Tailored physical activity prescription to address specific needs, abilities, personal circumstances and goals<br>23. Managing diverse needs within a group (different ages, physical abilities, symptom profiles)	experiential, behavioural, and physiological elements (1, 11)<br><br>g. Feedback processes: Processes through which current behaviour is compared against a particular standard (10, 14)<br><br>h. Environmental context & resources: Aspects of a person's situation or environment that discourage or encourage the behaviour (17, 18, 19, 20, 21, 22)<br><br>i. Norms: Attitudes held and behaviours exhibited by other people within a social group (15, 16, 23)<br><br>j. Beliefs about consequences: Perceptions about what will be achieved and/or lost by undertaking a behaviour, and probability that a behaviour will lead to a specific outcome (22, 23)	Instruction on how to perform behaviour (a, b, c)<br><br>Behaviour practice/rehearsal (esp. for exercise and stress-management techniques) (b, c)<br><br>Demonstration of behaviour (b,c)<br><br>Information about health consequences (a,e,j)<br><br>Social support (d,e)<br><br>Social comparison (d,e,g,i)<br><br>Habit formation (e,h)<br><br>Prompts and cues (self-established) (h)<br><br>Adding objects to the environment (h)<br><br>Restructuring the	Making physical activity sessions enjoyable, for example, by playing music, changing routine, playing games<br><br>Hospitals are associated with illness and traditional/disease-focused treatment, so the ideal venue may be outside of hospitals (e.g., in community venues). Around half of delivery sites will be community-based.<br><br>Participants are encouraged to improve their social support and build network of contacts around physical activity, e.g., by joining a walking group<br><br>Participants who are near the end of their core programme become an inspiration for those who are starting
Engaging in physical activity during and after the PERFORM programme	**Individual barriers**<br>24. Physical activity is not a priority (career responsibilities, employment responsibilities etc)	a. Knowledge: Awareness of the existence of something (28,29)		

Key behavioural targets	Modifiable barriers /enablers	Theoretical processes* (barriers addressed)	Behaviour change techniques* (processes addressed)	Specific strategies/intervention techniques (examples)
- physical activity for life<br><br>This includes ..<br><br>Researching the availability of physical activity opportunities in the local area<br><br>Trying out physical activity opportunities in the local area<br><br>Dealing with setbacks	25. Friends and family can discourage the person to engage in physical activity<br>26. Boredom (during physical activity)<br><br>**Individual enablers**<br>27. Making physical activity part of one's routine<br>28. Learning about the consequences of not exercising<br>29. Debunking the idea of physical activity as only being exercise sessions that happen in a gym<br>30. Developing trust in one's body, learning from experience and learning one's limits<br>31. Setting different types of goals (short/mid/long-term) – more focus on long-term wellbeing and quality of life<br>32. Learning from experience (noticing and acknowledging progress)<br>33. Involving support network<br>34. Access to different physical activity opportunities<br>35. Finding physical activity that one enjoys<br>36. Good experience of initial physical activity classes is crucial for the patient to continue<br>37. Having a buddy (a person or an app on the phone) as a way of generating motivation<br><br>**System-level barriers**<br>38. A lack of follow-up care/a lack of focus on engaging in physical activity in the long term<br>39. Conflicting advice from different healthcare professionals<br>40. Overreliance on medical procedures and medication (both by healthcare professionals and	e. Motivation: The impetus that gives purpose or direction to behaviour and operates at a conscious or unconscious level (24, 27)<br><br>d. Social influences: Interpersonal processes that can influence one's thoughts, feelings or behaviours (25, 27, 33, 36, 37, 44)<br><br>f. Emotion: A complex reaction pattern involving experiential, behavioural, and physiological elements (26, 35, 36)<br><br>k. Goals: Mental representations of outcomes or end states that an individual wants to achieve (31)<br><br>i. Environmental context & resources: Aspects of a person's situation or environment that discourage or encourage the behaviour (34, 38-43, 46)	social environment (d,h)<br><br>Identification of self as role model (e,f)<br><br>Focus on past success (c,e)<br><br>Individual tailoring of advice and support (not in BCW or taxonomy) (e, j)	Self-monitoring of progress<br><br>Using the Progress Tracker (above) to record learning and monitor progress, relating self-care changes to changes in physical and mental wellbeing<br>Using person-centred counselling techniques to deliver initial assessment, Health and Wellbeing sessions and discharge interviews<br><br>Facilitators encourage social interaction within the groups and inviting of family or friends to initial assessment, H&WB sessions and discharge appointment.<br><br>Some sessions include content encouraging reflection on past successes and personal strengths to boost

Key behavioural targets	Modifiable barriers /enablers	Theoretical processes* (barriers addressed)	Behaviour change techniques* (processes addressed)	Specific strategies/intervention techniques (examples)
	patients/carers)<br>41. Physical activity that you need to pay for<br><br>**System-level enablers**<br>42. Point of contact to ask questions about physical activity<br>43. Support for people to use technology to engage in ongoing physical activity<br>44. A clear process for handing over to carers at the end of the programme<br>45. Raising awareness of the benefits of physical activity amongst healthcare professionals – a strong, repeated message that physical activity is medicine<br>46. Signposting to different opportunities for engaging in ongoing and fun physical activities (e.g., walking groups, dance classes) and physical activity opportunities incorporating social experiences (e.g., team sports). Having easy access to physical activity equipment and locations.	j. Beliefs about consequences: Perceptions about what will be achieved and/or lost by undertaking a behaviour, and probability that a behaviour will lead to a specific outcome (28, 45)<br><br>c. Beliefs about capabilities: Beliefs about one's ability to successfully carry out a behaviour (30, 32)<br><br>g. Feedback processes: Processes through which current behaviour is compared against a particular standard (32)<br><br>b. Skill: An ability or proficiency acquired through practice (32, 33)<br><br>i: Norms: Attitudes held and behaviours exhibited by other people within a social group (39, 40, 45)		motivation and capability beliefs.<br><br>Eliciting and discussing expectations at the initial assessment /onboarding appointment

Key behavioural targets	Modifiable barriers /enablers	Theoretical processes* (barriers addressed)	Behaviour change techniques* (processes addressed)	Specific strategies/intervention techniques (examples)
Engaging in self-care behaviours (managing pain, managing stress, managing low mood, managing breathlessness, managing fatigue, eating healthily, sleeping well, managing medication) during and after the PERFORM programme<br><br>This includes …<br><br>Attending the Health and Wellbeing sessions<br><br>Testing self-care behaviours suggested during the Health and Wellbeing sessions<br><br>Noticing any associated improvements in quality of life<br><br>Evaluating which self-care behaviours suit the person's personality and lifestyle<br><br>Deciding which self-care behaviours can be easily integrated into one's routine and used long-term	**Individual barriers**<br>47. Experiencing a setback – becoming out-of-habit in using different self-care behaviour<br>48. Having too much on your plate – not having enough time and/or energy to engage in self-care behaviours<br><br>**Individual enablers**<br>49. Knowing what self-care behaviours to try and how to do them (e.g. breathing exercises)<br>50. Involving friends and family<br>51. Participants begin to concentre/focus on what they can do instead of what they cannot do<br>52. Participants share stories and testimonies – using social interaction/learning from different group participants/sharing and getting ideas<br>53. Engaging in physical activity for life (physical activity as a potent enabler for improving common symptoms experienced by people living with multiple long-term conditions)<br>54. Participants beginning to recognise benefits of engaging in suggested self-care behaviours, for example, staying independent<br>55. Utilising support network<br><br>**System-level barriers**<br>56. Lecture-style content may be heavy with medical/technical jargon and feel "schooly"<br><br>**System-level enablers**<br>57. Making the Health and Wellbeing sessions relevant to a wide audience – concentrate on self-help priorities of people living with multiple long-term conditions<br>58. Engaging name for the self-care sessions<br>59. An appropriate length of the Health and Wellbeing session (approx. 45 minutes + 15 minutes for [illegible]	a. Knowledge: Awareness of the existence of something (49, 52, 61, 62)<br><br>d. Social influences: Interpersonal processes that can cause oneself to change one's thoughts, feelings or behaviours (50, 52, 60)<br><br>c. Beliefs about capabilities: Beliefs about one's ability to successfully carry out a behaviour (47, 51)<br><br>h. Environmental context & resources: Aspects of a person's situation or environment that discourage or encourage the behaviour (56, 58, 59, 63, 64, 65, 66)<br><br>e. Motivation: Processes relating to the impetus that gives purpose or direction to behaviour and operates at a conscious or unconscious level (47, 48, 57, 58, 62)<br><br>j. Beliefs about consequences: Perceptions about what will be achieved and/or lost by undertaking a behaviour, and probability that a behaviour will lead to a specific outcome (53, 54, 57, 61, 62, 65)<br><br>[illegible]		

Key behavioural targets	Modifiable barriers /enablers	Theoretical processes* (barriers addressed)	Behaviour change techniques* (processes addressed)	Specific strategies/intervention techniques (examples)
Engaging in (attending and participating in) the PERFORM programme as a whole	68. Negative experiences from attending /not feeling engaged /feeling patronised<br>69. Access issues (transport, cost, time)<br>70. Patients have different expectations /preconceived ideas about what a rehabilitation programme will consist of.	d. Social influences: Interpersonal processes that can cause oneself to change one's thoughts, feelings or behaviours (69, 70)<br><br>h. Environmental context & resources: Aspects of a person's situation or environment that discourage or encourage the behaviour (69)<br><br>j. Beliefs about consequences: Perceptions about what will be achieved and/or lost by undertaking a behaviour, and probability that a behaviour will lead to a specific outcome (70)<br><br>l. Attitude towards the behaviour: General evaluations of the behaviour on a scale ranging from negative to positive (68, 70)<br><br>m. Building empathy /person-centred delivery style (68)		

* Labels and definitions are taken from the Theory and Techniques Tool where possible[31]
