# Supplementary material for: Co-design and development of a Personalised Exercise-based Rehabilitation and self-management programme FOR people with Multiple long-term conditions: The PERFORM intervention
Source: J Multimorb Comorb. 2025 Sep 18;15:26335565251367326. doi: 10.1177/26335565251367326 (PMC12446826; doi:10.1177/26335565251367326)
Supplement: Supplemental Material - Co-design and development of a personalised exercise-based rehabilitation and self-management programme for people with multiple long-term conditions: The PERFORM intervention [file sj-pdf-5-cob-10.1177_26335565251367326.pdf]

. Supplementary Table S2: Behavioural targets, associated barriers and enablers, theoretical processes of change and strategies to effect change for healthcare professionals in the PERFORM intervention.

Key behavioural targets	Modifiable barriers /enablers	Theoretical process (barriers addressed)*	Behaviour change techniques	Specific strategies/intervention techniques[$]
**Deliver the PERFORM programme as intended:**<br><br>**a) Initial assessment**<br>Assess individual needs and concerns (esp. about exercise) using the assessment template questions.<br>Identify any requirements for exercise adaptation. Provide condition-specific information where needed. Address any patient queries about the programme. Use a mixed communication style (asking both closed and open questions to elicit information, but more open questions, reflective listening/ask-tell-discuss to discuss any barriers and improve patient understanding). Assess exercise capacity using an appropriate cardiorespiratory fitness test.<br><br>**b) Group-based exercise sessions**<br>Provide a tailored exercise prescription, based initially on the individual's cardiorespiratory fitness test result (where available).<br>Monitor for working at moderate intensity and safe/correct performance of exercises during the supervised group sessions. Monitor progress and progress/regress exercise intensity or effort to achieve moderate intensity. Encourage patient self-monitoring and adjustment of effort using the PERFORM Effort Scale (or similar). Use a mixed communication style (as above).<br><br>**c) Home-based exercise prescription**<br>Agree an individually-tailored home-based exercise programme for each patient.<br>Monitor progress (through checking the Progress Tracker) and progress/regress exercise intensity or effort to achieve moderate intensity of activity.<br>Encourage patient self-monitoring and adjustment of effort using the PERFORM Effort Scale (or similar).<br>Use a mixed communication style (as above).	**Individual barriers**<br>1. Lacking the skills or confidence to work with a wider set of patients in terms of more diverse health conditions.<br>2. Fear of exercising patients with more complex needs, or not knowing enough about specific long-term conditions.<br>3. A lack of experience of delivering group-based educational sessions.<br><br>**Individual enablers**<br>4. Understanding the difference between the PERFORM programme and traditional rehabilitation programmes (e.g., to be clear/transparent that disease-specific education will be outside of the remit of the intervention).<br>5. Understanding the purpose and value of the PERFORM programme.<br>6. Understanding what the intervention requires of them and how to deliver	a. Knowledge: Awareness of existence of something<br>(2, 4, 5, 6, 7, 9, 11)<br><br>b. Skill: An ability or proficiency acquired through practice<br>(1, 2, 6, 7, 8)<br><br>c. Beliefs about capabilities /one's ability to successfully carry out a behaviour<br>(1, 2, 3, 7, 8)<br><br>j. Beliefs about consequences: (perceptions about what will be achieved or lost by undertaking a behaviour, as well as the probability that a behaviour will lead to a specific outcome)<br>(2, 5)<br><br>h. Environmental context &	Information about health consequences (during the initial training and the refresher training)(a,j)<br><br>Instruction on how to perform behaviour (during the initial and refresher training)<br>(a,b,c)<br><br>Behavioural practice/rehearsal<br>(b,c)<br><br>Demonstration of behaviour (during the initial and refresher training)<br>(b,c)<br><br>Problem solving (during initial training and refresher training)<br>(b,c,h)<br><br>Feedback on behaviour (via self-assessment of intervention fidelity and online	**Good quality training:**<br>Training to emphasise and discuss expected outcomes of the programme for patients (better quality of life, aerobic capacity, physical and mental wellbeing).<br><br>Provide opportunities to practice skills, reflect on personal practice (self-reflection tools) and to share ideas and experiences of delivery with other trainees (social media based community of practice).<br><br>Demonstration videos to model good practice /intended delivery style and how to deliver /support key BCTs (reviewing progress, action-planning etc).<br><br>Covering contraindications for exercising certain long-term conditions (but not to "overeducate" trainees).<br><br>Implications of delivering exercise sessions for

**d) Health and Wellbeing sessions**<br>Use a person-centred communication style.<br>Deliver all the intended educational content (all slides) and interactive educational activities in each session.<br>Encourage and support action-planning and discussion about individual plans of action relating to the content/ideas in each session.<br>Review progress with planned changes in self-care behaviours.<br>Support and encourage problem-solving to overcome any barriers in changing self-care behaviours.<br>Use the Ask-Tell-Discuss approach (as indicated in the session slides/session notes) to build patient understanding of and motivation for self-care.<br>Encourage open discussion of the topics delivered in each session.<br>Use reflective listening or other techniques to keep the discussion focused on the intended topic and avoid lengthy deviations.<br>Acknowledge/normalise psychological consequences of living with LTCs and help patients find ways to address them.<br><br>**e) Discharge appointment**<br>Use a person-centred communication style (as above).<br>Review progress made since the start of the programme.<br>Discuss/agree plans for ongoing exercise and other key self-care behaviours for the individual patient.<br>Relapse prevention: identify and try to address any possible barriers to ongoing exercise and key self-care behaviours.<br>Use the discharge appointment template questions.<br>Identify and explore any remaining concerns or patient queries about their ongoing self-care.<br>Assess exercise capacity using the same cardiorespiratory fitness test as used for initial assessment.<br><br>**f) Maintenance/review sessions**<br>Use a person-centred communication style (as above).	the PERFORM programme (e.g., striking a balance between giving general and specific (albeit wellbeing) advice to patients).<br>7. Potentially disruptive (or facilitative) group dynamics.<br>8. Efficiency (time, resources, skills) in delivering individual assessment and tailoring.<br>9. Developing a database of signposting opportunities available in the area requires significant resources<br><br>**System-level barriers**<br>10. Too much focus on managing individual conditions rather than exercising and broader determinants of wellbeing.<br>11. A lack of access to specialist advice about the safety of exercise for patients with certain health conditions.<br><br>**System-level enablers**<br>12. Access to a multisystem/MDT	resources: Aspects of a person's situation or environment that discourage or encourage the behaviour<br>(8, 10, 11, 12)<br><br>i. Norms: Attitudes held and behaviours exhibited by other people within a social group<br>(10, 12, 13)<br><br>n. Leadership support (not in the BCT taxonomy): Material support (e.g. equipment, space, session-delivery slides), organising access to specialist advice, affirmation by senior managers, establishing normative beliefs, practices and standards, advocating changes to other leaders /managers within the system.<br>(12, 13)	community of practice) (b,c)<br><br>Monitoring of (patient) outcomes of behaviour (through direct observation, audit data and patient outcomes) (c,k)<br><br>Reduce negative emotions (through practice and becoming more confident at intervention delivery) (b,c,j)<br><br>Engaging key opinion leaders /service managers in the co-design and setup of the revised service model (n,o).	different conditions and how to do it safely (understanding the basic exercise pathophysiology).<br><br>Emergency procedures, for example, how to respond if a patient collapses or has a breathing crisis during exercise.<br><br>Red/orange exercise flags for specific conditions to be identified and adjusted for at the initial assessment and each exercise session.<br><br>Setting up expectations early on – giving patients confidence about the intervention and the healthcare professionals delivering it.<br><br>Being crystal clear about the scope /role of service providers and the intervention (healthcare professionals to be permitted not to know everything and use signposting for more complex issues/questions).<br><br>Managing patient disease-specific questions – might require more 1-1 input (for

Review progress with and support maintenance of exercise and/or physical activity since leaving the core supervised programme.<br>Review progress with and support maintenance of other self-care activities since leaving the core supervised programme.<br>Support and encourage problem-solving to overcome any barriers in changing self-care behaviours.<br>Use reflective listening or other techniques to keep the discussion focused on the intended topic.<br>Acknowledge/normalise psychological consequences of living with LTCs and help patients find ways to address them.<br><br>**g) Caregiver involvement**<br>Encourage involvement of any friends or family who may be present during initial assessment, Health and Wellbeing sessions, discharge appointment, or maintenance sessions.<br>Encourage involvement of any friends or family who are not present in the sessions to support intended changes in self-care.	approach – having a place where healthcare professionals can discuss more complex patients.<br>13. Champions advocating for multimorbidity /more holistic care /going against the grain of the disease-based model which is currently dominant in the NHS.	o. Engaging service champions (not in BCT taxonomy): Engaging key opinion leaders /service managers in the co-design and setup of the revised service model. (12, 13)		example by having a private conversation), signposting to the relevant healthcare professionals or organisations.<br><br>**Quality support materials:**<br><br>Build in key messages to the structure of the intervention (content and sequencing of slides for the H&WB sessions) – e.g. threading of key messages such as “exercise is medicine” and “make changes you can live with”.

* Labels and definitions from the Theory and Techniques Tool where possible[31]

These are examples rather than a full /comprehensive list
